# Vaginal Leiomyoma: A Case Report

**DOI:** 10.31729/jnma.6180

**Published:** 2021-05-31

**Authors:** Miki Shah, Rachana Saha, Niranjan KC

**Affiliations:** 1 Department of Obstetrics and Gynaecology, Kathmandu Medical College and Teaching Hospital, Sinamangal, Kathmandu, Nepal; 2 Kathmandu Medical College and Teaching Hospital, Sinamangal, Kathmandu, Nepal

**Keywords:** *case report*, *leiomyoma*, *vagina*

## Abstract

Leiomyomas are common benign tumors of the uterus, affecting 20-30% of women of reproductive age group. But vaginal leiomyomas remain an uncommon entity with only about 300 reported cases. The first case was described in 1733. Only a few cases have been reported in Nepal to date. Tumors are thought to arise from Mullerian smooth muscle cells in the sub-epithelium of the vagina. Vaginal leiomyomas are usually situated in the anterior vaginal wall. Here, we report a case of a 48-year-old multipara who presented the outpatient department with the ultrasonographic report showing multiple uterine fibroids but was asymptomatic. A physical examination showed a mass in the right vaginal wall. Pervaginal removal of the tumor was performed and subsequent histopathology revealed a vaginal leiomyoma. Removal of the tumor by the vaginal route, wherever possible, with the subsequent histopathological examination, appears to be the optimum management plan.

## INTRODUCTION

Vaginal tumors are rare and include papilloma, hemangioma, mucus polyp, and rarely leiomyoma. Vaginal leiomyomas remain an uncommon entity with only about 300 reported cases since the first detected case back in 1733 by Denys de Leyden.^1^ So far, only a few cases has been reported in Nepal. These tumors arise most commonly from the anterior vaginal wall causing varied clinical presentations. They may or may not be associated with leiomyomas elsewhere in the body. We report a case of a 48-year-old multipara who presented the outpatient department with the ultrasonographic report showing multiple uterine fibroids but was asymptomatic.

## CASE REPORT

A 48-year-old para three lady was presented to the outpatient department (OPD) with no complaint but a routine ultrasonography (USG) showed multiple fibroid uterus, largest measuring 1 x 1 cm at the fundus. On per speculum examination, a mass of 4 x 2 x 2 cm was seen on right vaginal wall. On Per-vaginal examination, uterus was bulky and fornices were free. A firm mass with a smooth surface was felt in right lateral vaginal wall. Therefore, a provisional diagnosis of vaginal leiomyoma was made. The patient was counseled for surgery. Informed consent was taken for photography and publication.

**Figure 1.**
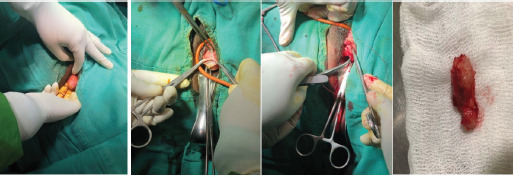
Intraoperative and postoperative pictures demonstrating removal of leiomyoma via vulvovaginal incision and its closure.

Removal of mass was planned vaginally. A foley's catheter was introduced into the urethra. Vaginal mucosa was separated from the mass and the mass was enucleated which was sent for histopathological examination. Redundant tissue was cut and approximated. Gross examination of the mass revealed a 4 × 2 × 2cm, firm solid mass with a smooth outline. The cut section showed a whorled appearance (Figure 1).

**Figure 2.**
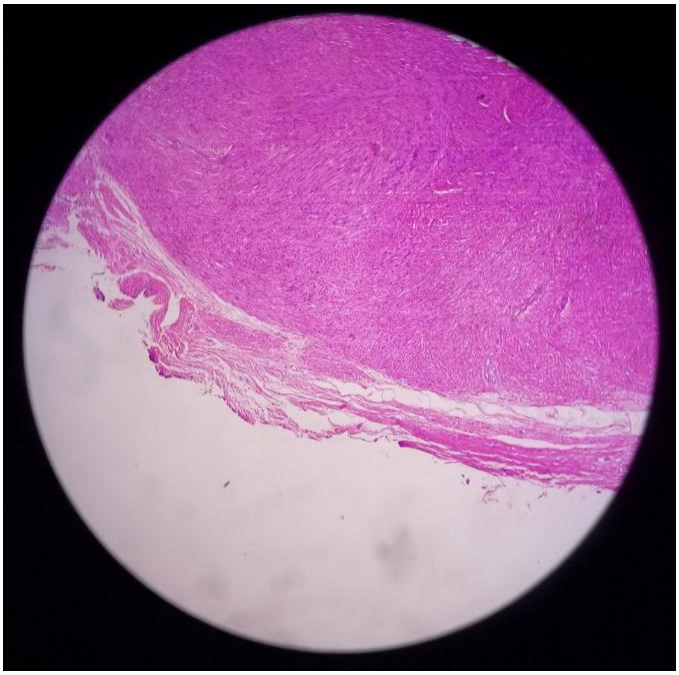
Vaginal tumor showing dense aggregation of spindle-shaped cells.

Microscopy revealed a circumscribed mass composed of smooth muscles arranged in intersecting bundles and fascicles without atypia, mitosis, and necrosis. Few dilated and congested vessels were also seen (Figure 2).

Her post-operative period was uneventful and she was discharged on the same day. On the 7th post-operative day, the patient was followed up at OPD with no complaints and the operation site was healthy.

## DISCUSSION

Leiomyomas in the female genital tract are common in the uterus and to some extent in the cervix followed by the round ligament, uterosacral ligament, ovary, and inguinal canal.^1^ Occurrence in the vagina is very rare. These tumors arise most commonly from the anterior vaginal wall causing varied clinical presentations.

They may or may not be associated with leiomyomas elsewhere in the body. Vaginal leiomyomas are commonly seen in the age group ranging from 35 to 50 years and are reported to be more common among Caucasian women.^2^ They usually occur as a single, well-circumscribed mass arising from the midline anterior wall and less commonly, from the posterior and lateral walls.^1,3,4^ They may be asymptomatic but may give rise to cyclic urinary retention, dyspareunia, gluteal swellings with vaginal purulent discharge, obstruction in the birth passage if along with pregnancy, or simply a feeling of mass in vagina.^5,6^ These tumors can be intramural or pedunculated and solid as well as cystic. Usually, these tumors are single, benign, and slow-growing, but sarcomatous transformation has been reported.^7^

Surgical removal of the tumor through a vaginal approach, preferably with urethral catheterization to protect the urethra during surgery, is usually the treatment of choice.^5^ In the case of large tumors, however, an abdominoperineal approach is preferred. Histopathological confirmation is the gold standard of diagnosis and also beneficial to rule out any possible focus of malignancy.
